# Ameliorative Effects of Citrus Unshiu Peel Extract in Cellular and Mouse Models of Pelizaeus–Merzbacher Disease

**DOI:** 10.3390/ijms27198732

**Published:** 2026-09-30

**Authors:** Miyu Yamamoto, Hideya Sakaguchi, Moeri Yagi, Yuta Muraki, Akinori Nishi, Hideji Yako, Junji Yamauchi, Yuki Miyamoto

**Affiliations:** 1 Department of Precision Medicine and Drug Discovery, National Research Institute for Child Health and Development, Tokyo 157-8535, Japan; 2 Tsumura Advanced Technology Research Laboratories, Tsumura & Co., Ibaraki 300-1192, Japanmuraki_yuuta@mail.tsumura.co.jp (Y.M.);; 3 Laboratory of Molecular Neuroscience and Neurology, Tokyo University of Pharmacy and Life Sciences, Tokyo 192-0392, Japan; hyako@toyaku.ac.jp

**Keywords:** PMD, HLD1, cellular effects, mouse, Chinpi

## Abstract

Hypomyelinating leukodystrophy 1 (HLD1), also known as Pelizaeus–Merzbacher disease (PMD), is a severe X-linked hereditary hypomyelinating/demyelinating disorder caused primarily by genomic duplications or multiplications that lead to the increased expression of proteolipid protein 1 (PLP1). Aberrant PLP1 expression impairs oligodendrocyte differentiation and disrupts myelin formation in the central nervous system (CNS). Despite decades of research, effective disease-modifying therapies remain unavailable. In the present study, we investigated the potential of Chinpi, the Citrus Unshiu Peel extract commonly utilized in traditional herbal medicine and reported to protect cells from various cellular stresses, using both in vitro cellular and in vivo animal models of PMD. Using an FBD-102b oligodendrocyte precursor model cell line engineered to overexpress PLP1, we demonstrated that treatment with Chinpi significantly ameliorates critical defects in morphological differentiation and successfully restores the expression of major myelin proteins. Furthermore, oral administration of Chinpi to PLP1 transgenic mice improves myelin protein expression in the brain and significantly alleviates progressive motor incoordination, as assessed by behavioral tests. Taken together, these findings suggest that Chinpi exerts protective effects against PLP1-induced morphological differentiation defects and subsequent reductions in myelin protein expression, highlighting its potential as a recovery approach for PMD.

## 1. Introduction

Neurons transmit information through axons that extend from their cell bodies. In the central nervous system (CNS), neuronal axons are wrapped by multilayered myelin sheaths [1,2,3,4,5]. During myelination, oligodendrocytes undergo extensive plasma membrane expansion to generate the multilamellar myelin structure produced by these CNS glial cells [1,2,3,4,5]. Gaps between adjacent myelin sheaths, termed nodes of Ranvier, enable saltatory conduction, thereby greatly increasing the speed of nerve impulse propagation. In addition to facilitating rapid electrical conduction, oligodendrocytes metabolically support neurons by supplying glycolysis-derived lactate as an energy source through axon–glia interactions [6,7]. Consequently, defective myelin formation or myelin damage caused by physiological or physical stresses contributes to severe neurodegenerative diseases [1,8,9,10].

Genetic hypomyelinating disorders are collectively referred to as hypomyelinating leukodystrophies (HLDs). These rare conditions have an estimated prevalence of approximately 1 in 250,000 individuals [11,12,13,14,15]. HLD1, also known as Pelizaeus–Merzbacher disease (PMD), is an X-linked disorder characterized by hypomyelination and/or demyelination in the CNS [11,12,13,14,15]. PMD is caused by mutations in the gene encoding proteolipid protein 1 (PLP1) and is frequently associated with increased PLP1 expression resulting from gene duplication or multiplication.

PLP1 is a major four-transmembrane myelin protein, accounting for 30 to 50% of the total protein in CNS myelin [1,2,3,4,5]. A major mechanism underlying PMD is that aberrant PLP1 expression induces severe endoplasmic reticulum (ER) stress, which activates the unfolded protein response (UPR) in oligodendrocytes. UPR activation is thought to impair oligodendrocyte differentiation and result in incomplete and/or defective myelin sheath formation [14,15,16]. Clinically, patients with PMD exhibit nystagmus and developmental delay soon after birth, with progressive deterioration leading to spastic quadriplegia and cerebellar ataxia [13,15,17]. Despite the severity of these symptoms, current treatments remain limited to symptomatic therapies, including anticonvulsants and muscle relaxants, and no curative therapy is currently available [13,15,17]. Although preclinical and clinical trials have increased in recent years, effective treatments remain limited and face significant challenges [13,15,17].

Herein we describe the effects of Chinpi, the Citrus Unshiu Peel extract, in in vitro and in vivo models of PMD. Chinpi is commonly used as an ingredient in herbal medicine [18]. It is known to possess anti-oxidant and anti-inflammatory properties and exert neuroprotective effects in neurological disorders associated with organelle stress and inflammation [19,20,21,22]. We used oligodendroglial FBD-102b cells, which morphologically and biochemically reproduce oligodendrocyte differentiation [23,24], and overexpressed PLP1 to model increased PLP1 expression in cells [25,26]. Using this system, we found that Chinpi ameliorated PLP1 overexpression-induced defects in morphological differentiation and myelin protein expression. Furthermore, administration of Chinpi to PLP1 transgenic (Tg) mice, an in vivo model of PMD [27], restored myelin protein expression and alleviated motor incoordination. These findings suggest that Chinpi may provide an ameliorative effect for PMD through dietary or pharmacological interventions.

## 2. Results

### 2.1. PLP1 Overexpression Exhibits Decreased Morphological Differentiation, Which Is Restored by Chinpi

**Figure 1:**
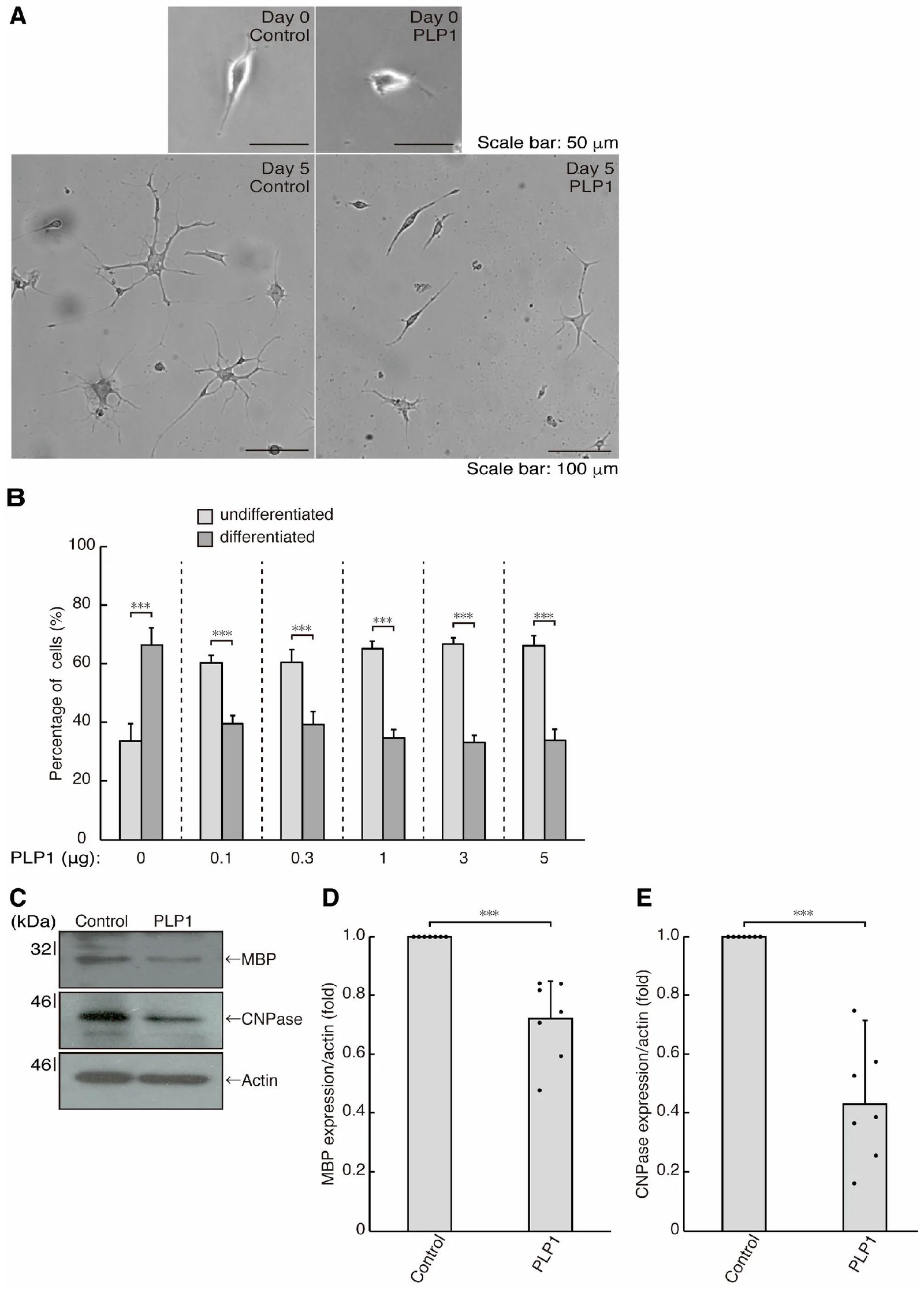
Overexpression of PLP1 decreases morphological differentiation. (**A**,**B**) FBD-102b cells were transfected with a plasmid encoding PLP1 (0.1–5 μg) or empty vector and differentiated for 5 days. Representative images of control and 3 μg transfected cells are shown. The number of undifferentiated and differentiated cells was counted, and their percentages were quantified (***, *p* < 0.001; Student’s *t*-test; averaging the values from three fields of view per dish and treating the experiment as having *n* = 3). (**C**–**E**) Cell lysates prepared 5 days after induction of differentiation were subjected to immunoblotting using antibodies against MBP, CNPase, and control actin proteins. Quantification of each immunoreactive band intensity normalized to the actin loading control is shown (***, *p* < 0.001; Student’s *t*-test; *n* = 7 blots). Individual values are plotted as closed circles.

To examine the effect of PLP1 expression on oligodendroglial morphological differentiation, we transfected FBD-102b cells with a plasmid encoding PLP1 and cultured the cells under differentiation conditions for 0 or 5 days. Following induction of differentiation, approximately 65% of control vector-transfected cells exhibited differentiated phenotypes characterized by multiple elongated branches extending from the cell bodies (Figure 1A). In contrast, PLP1-transfected cells showed significantly decreased morphological differentiation in a dose-dependent manner (Figure 1A,B).

We next examined the expression levels of oligodendroglial differentiation and myelination markers in control- and PLP1-transfected cells. After induction of differentiation, the expression levels of MBP and CNPase were markedly reduced in PLP1-transfected cells compared with control cells, whereas the expression level of the control actin was comparable between the two groups (Figure 1C–E).

Because ER stress signaling is enhanced in experimental PMD model cells [4,15,16], including activation of the eIF2alpha and C/EBP homologous protein (CHOP) pathways, we investigated whether overexpressed PLP1 induced similar responses. Phosphorylation of eIF2alpha was detected in PLP1-transfected cells (Figure S1A,B). Similarly, CHOP expression was increased in cells overexpressing PLP1 (Figure S1C,D), indicating that ER stress signaling was stimulated in the PLP1-expressing cells.

Since oligodendroglial differentiation is closely associated with Akt signaling [28,29], we further assessed the phosphorylation status of Akt kinase at the Ser-473 position, which reflects its activation state [28,29]. Akt phosphorylation increased following differentiation in control cells. In contrast, PLP1 transfection markedly suppressed Akt phosphorylation levels (Figure S2A,B). Collectively, these results suggest that PLP1 expression suppresses oligodendroglial morphological differentiation, accompanied by reduced expression of differentiation markers and decreased Akt activation.

**Figure 2:**
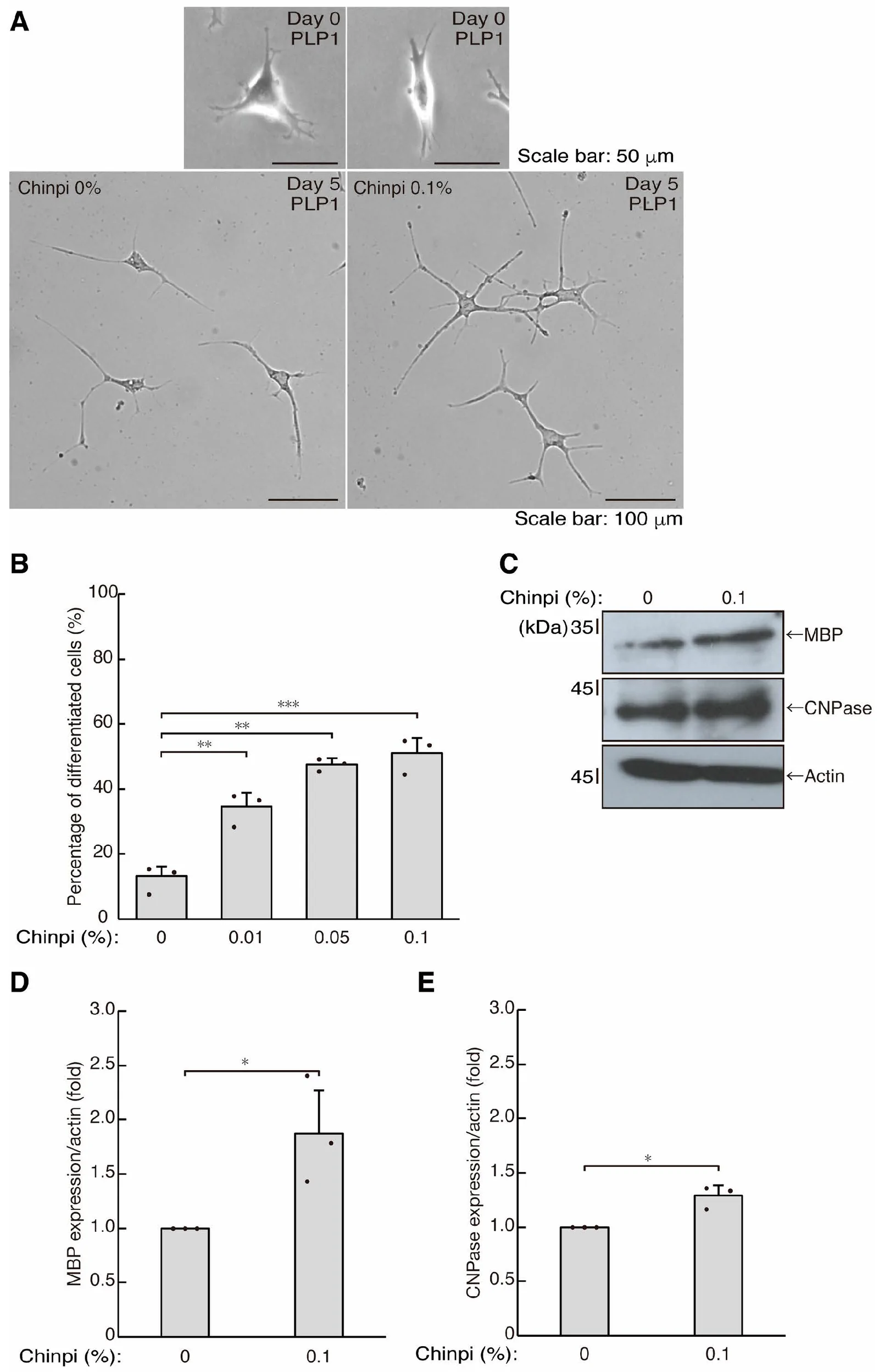
Treatment with Chinpi extract restores PLP1-induced morphological abnormalities. (**A**,**B**) FBD-102b cells were transfected with a plasmid encoding PLP1 and differentiated for 5 days in the presence or absence of Chinpi extract (0–0.1%). Representative images of cells treated with 0 and 0.1% Chinpi are shown. The number of undifferentiated and differentiated cells was counted, and their percentages were quantified (***, *p* < 0.001, **, *p* < 0.01; one-way ANOVA; averaging the values from three fields of view per dish and treating the experiment as having *n* = 3). Individual values are plotted as closed circles. (**C**–**E**) Cell lysates prepared 5 days after induction of differentiation were subjected to immunoblotting using antibodies against MBP, CNPase, and control actin proteins. Quantification of each immunoreactive band intensity, normalized to the actin loading control, is shown (*, *p* < 0.05; Student’s *t*-test; *n* = 3 blots). Individual values are plotted as closed circles.

Next, we investigated whether Chinpi, dried mandarin orange peel, could restore the reduction in differentiation induced by PLP1 transfection. Treatment with Chinpi extract ameliorated the morphological phenotypes caused by PLP1 in a concentration-dependent manner (Figure 2A,B). Consistently, the PLP1-induced reductions in MBP and CNPase expression were also reversed by Chinpi treatment (Figure 2C–E).

We further examined whether Chinpi could suppress the activation of the ER stress pathway induced by PLP1 overexpression. Treatment with Chinpi reduced both the phosphorylation levels of eIF2alpha and CHOP expression (Figure S3A–D), suggesting that Chinpi attenuates PLP1-induced ER stress.

In addition, Chinpi restored Akt phosphorylation levels in PLP1-transfected cells (Figure S4A,B).

Taken together, these results indicate that Chinpi alleviates PLP1-induced morphological abnormalities at the cellular level.

### 2.2. Chinpi Restores Myelin Marker Proteins in PLP1 Tg Mice

**Figure 3:**
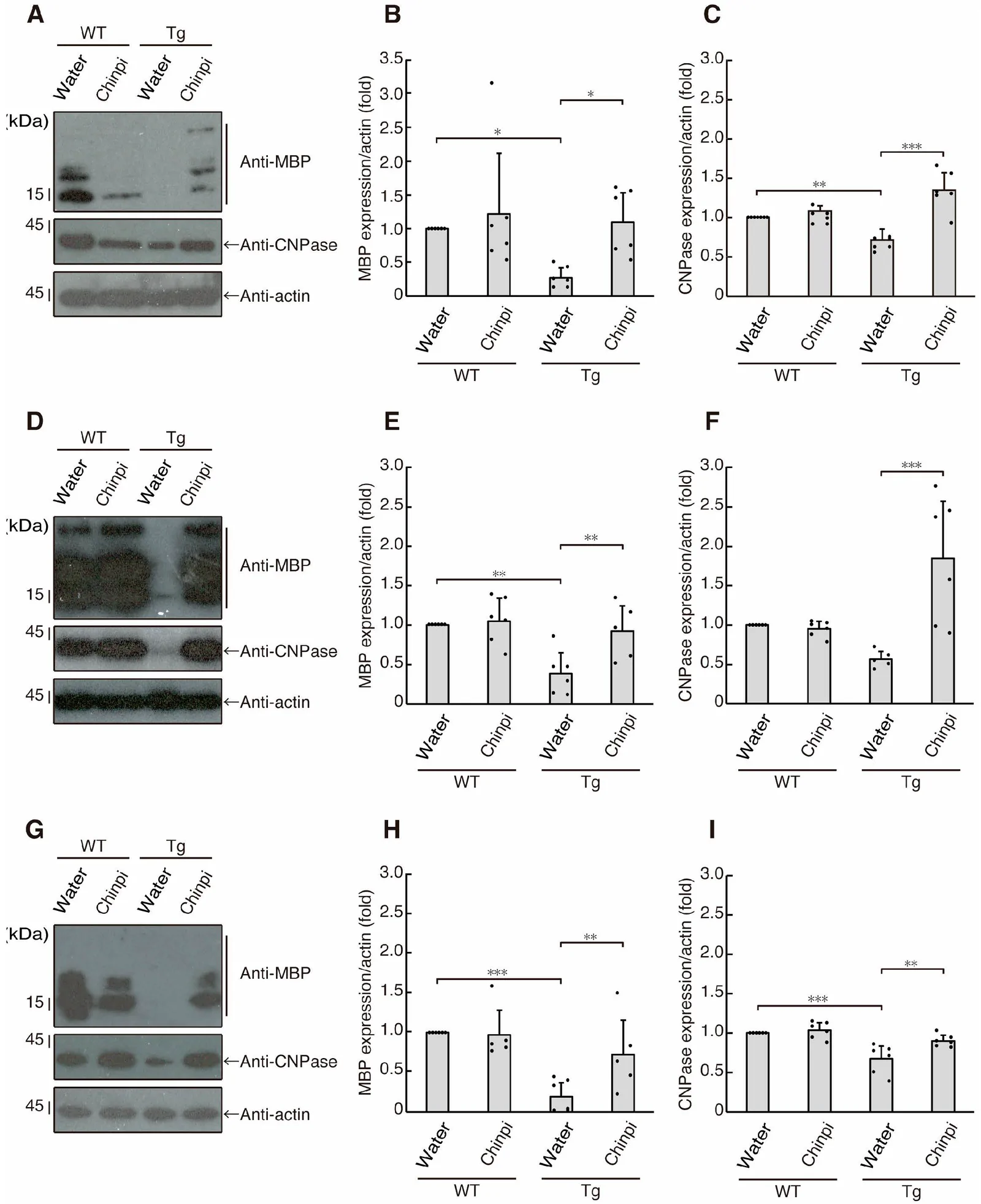
Treatment with Chinpi extract restores the reduction in myelin marker proteins observed in PLP1 Tg mice. (**A**–**I**) Cortex (**A**–**C**), corpus callosum (**D**–**F**), and cerebellum (**G**–**I**) tissue lysates of PLP1 Tg and littermate wild-type treated with water or Chinpi were subjected to immunoblotting with antibodies against MBP, CNPase, and control actin proteins. Quantification of each immunoreactive band intensity, normalized to the actin loading control, is shown (**B**) *p* = 0.2689; two-way ANOVA followed by Bonferroni’s test: *p* = 0.0462 water. WT vs. water. Tg, *p* = 0.0201 water. Tg vs. Chinpi. Tg, (**C**) *p* < 0.01; two-way ANOVA followed by Bonferroni’s test: *p* = 0.0197 water. WT vs. water. Tg, *p* < 0.001 water. Tg vs. Chinpi. Tg, (**E**) *p* = 0.1173; two-way ANOVA followed by Bonferroni’s test: *p* = 0.0015 water. WT vs. water. Tg, *p* = 0.0042 water. Tg vs. Chinpi. Tg, (**F**) *p* < 0.01; two-way ANOVA followed by Bonferroni’s test: *p* = 0.1410 water. WT vs. water. Tg, *p* < 0.001 water. Tg vs. Chinpi. Tg, (**H**) *p* = 0.0804; two-way ANOVA followed by Bonferroni’s test: *p* < 0.001 water. WT vs. water. Tg, *p* = 0.0115 water. Tg vs. Chinpi. Tg, (**I**) *p* = 0.1048; two-way ANOVA followed by Bonferroni’s test: *p* = 0.0001 water. WT vs. water. Tg, *p* = 0.0021 water. Tg vs. Chinpi. Tg; *n* = 5–7 blots collected from independent pairs of littermates. Individual values are plotted as closed circles. ***, *p* < 0.001, **, *p* < 0.01, *, *p* < 0.05.

Since reduced expression levels of myelin marker proteins were observed in PLP1-overexpressing cells, we next examined whether similar changes occurred in PLP1 Tg mice. Although PLP1 Tg mice are generally behaviorally normal during early developmental stages, some animals develop ataxia and seizures at 3 to 4 months of age, and a subset dies prematurely at approximately 6 months of age [27]. Therefore, 4-month-old PLP1 Tg mice were used for the following experiments. Compared with wild-type littermate controls, PLP1 Tg mice exhibited reduced expression levels of MBP and CNPase in the cerebral cortex, corpus callosum, and cerebellum (Figure 3A–I), whereas control actin expression remained unchanged.

We next investigated whether Chinpi could ameliorate the reduction in expression levels of myelin marker proteins observed in PLP1 Tg mice. The expression levels of MBP and CNPase in PLP1 Tg mice were restored with Chinpi treatment to levels comparable to those in control mice (Figure 3A–I). These findings suggest that Chinpi ameliorates reduced myelin marker protein expression in PLP1 Tg mice.

### 2.3. Chinpi Improves Motor Function in PLP1 Tg Mice

To determine whether the molecular abnormalities observed in PLP1-transfected cells and PLP1 Tg mice were associated with motor and functional deficits, we evaluated the behavioral phenotypes of PLP1 Tg mice.

**Figure 4:**
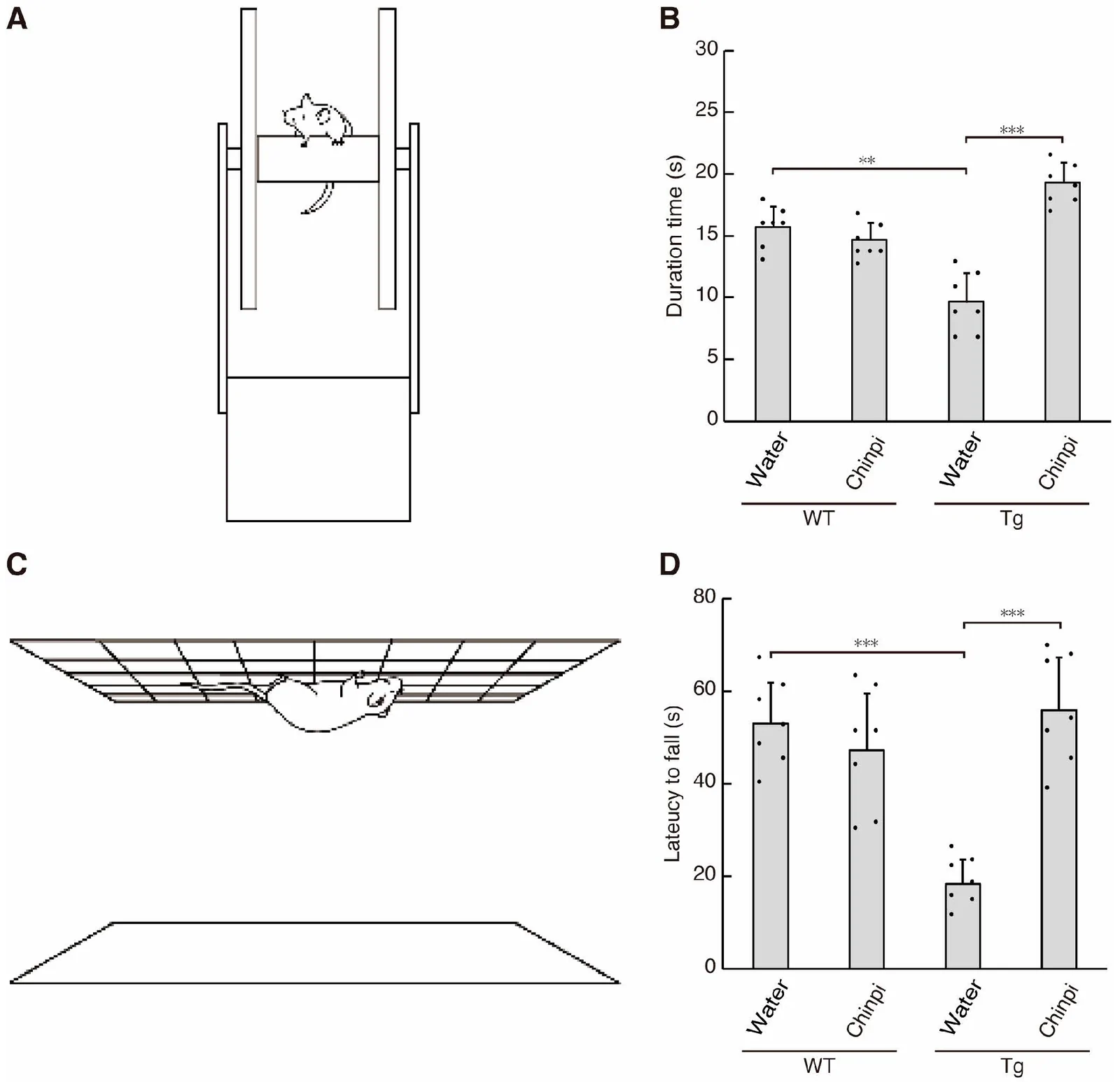

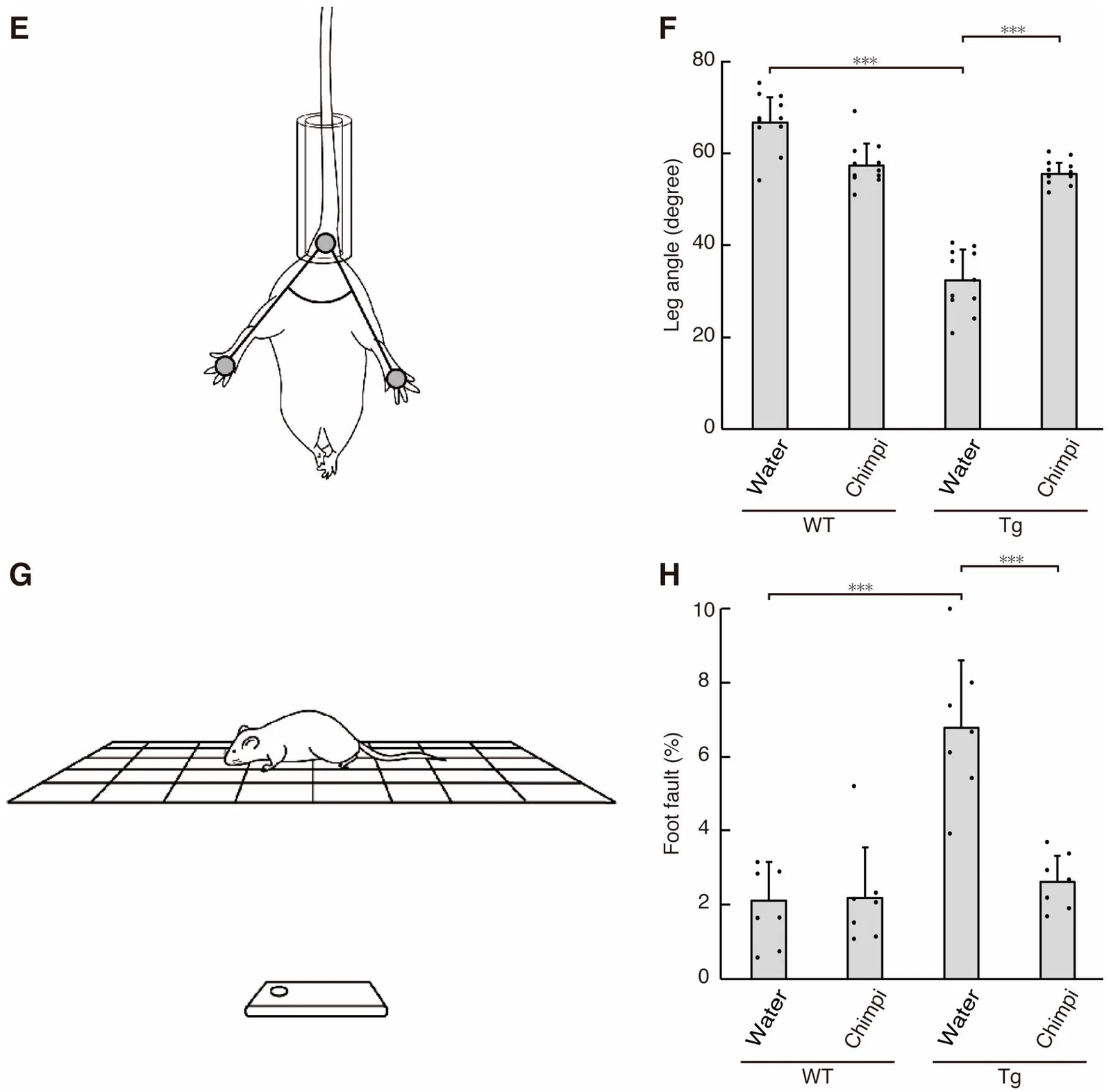
Chinpi extract restores motor dysfunction observed in PLP1 Tg mice. (**A**) Schematic of the rotarod test is shown. (**B**) Latency to fall from the rotating rod in PLP1 Tg and littermate wild-type treated with water or Chinpi is shown (*p* < 0.01; two-way ANOVA followed by Bonferroni’s test: *p* = 0.0048 water. WT vs. water. Tg, *p* < 0.001 water. Tg vs. Chinpi. Tg; *n* = 7 mice, independent pairs of littermates). (**C**) Schematic of the wire hang test is shown. (**D**) Latency to fall from the wire in PLP1 Tg and littermate wild-type treated with water or Chinpi is shown (*p* < 0.001; two-way ANOVA followed by Bonferroni’s test: *p* < 0.001 water. WT vs. water. Tg, *p* < 0.001 water. Tg vs. Chinpi. Tg; *n* = 7 mice, independent pairs of littermates). (**E**) Schematic of the tail suspension test is shown. (**F**) Hindlimb angle in PLP1 Tg and littermate wild-type treated with water or Chinpi is shown (*p* < 0.001; two-way ANOVA followed by Bonferroni’s test: *p* < 0.001 water. WT vs. water. Tg, *p* < 0.001 water. Tg vs. Chinpi. Tg; *n* = 11 mice, independent pairs of littermates). (**G**) Schematic of the grid-walking test is shown. (**H**) Hindlimb foot-fault rate in PLP1 Tg and littermate wild-type treated with water or Chinpi is shown (*p* < 0.01; two-way ANOVA followed by Bonferroni’s test: *p* < 0.001 water. WT vs. water. Tg, *p* < 0.001 water. Tg vs. Chinpi. Tg; *n* = 7 mice, independent pairs of littermates). ***, *p* < 0.001, **, *p* < 0.01.

First, mice were subjected to accelerating rotarod and wire hang tests to assess motor coordination, balance, and muscle strength (Figure 4A,C). The latency to fall from the rotating rod or wire was significantly shorter in PLP1 Tg mice than in littermate control mice. Notably, Chinpi treatment significantly improved performance in both behavioral tests (Figure 4B,D).

Next, we performed a tail suspension test by measuring the angle formed between the bilateral hindlimbs (Figure 4E). Most littermate control mice maintained widely extended hindlimbs, whereas PLP1 Tg mice consistently exhibited hindlimb clasping with markedly narrower angles throughout the test. Chinpi treatment significantly increased the hindlimb angle, indicating improvement of the clasping phenotype (Figure 4F).

We also conducted a grid-walking test to assess sensorimotor function and motor coordination. In this assay, mice were allowed to walk on an elevated wire grid for 5 min, and the number of hindlimb slips was quantified (Figure 4G). The foot-fault rate was significantly higher in PLP1 Tg mice than in littermate control mice, whereas Chinpi treatment significantly reduced the foot-fault rate (Figure 4H).

Collectively, these results demonstrate that PLP1 Tg mice exhibit neurological and motor deficits and that Chinpi effectively ameliorates these behavioral abnormalities.

## 3. Discussion

The present study demonstrates, for the first time to our knowledge, that Chinpi, a traditional herbal medicine, exerts protective effects in both in vitro and in vivo models of PMD. PMD is a severe and progressive neurodegenerative disorder for which no disease-modifying therapies are currently available [13,15,17]. Our findings therefore highlight the potential of Chinpi to mitigate PMD pathogenesis at both the cellular and mouse levels.

Recent therapeutic strategies for PMD in the mid-2020s can be broadly categorized into three or four major groups, including approaches with encouraging preclinical outcomes, those with limited or inconclusive efficacy thus far, and therapies under active development [13,15,17]. These include: (1) direct suppression of the causative PLP1 gene, such as antisense oligonucleotides; (2) modulation of the integrated stress response mediated by stress-responsive kinases activated under various conditions, including ER stress; (3) amelioration of metabolism and lipid trafficking; and (4) transplantation of neural lineage precursor or stem cells [30,31,32,33,34,35]. The effects of Chinpi identified in the present study may share mechanical similarities with the second category. Aberrant PLP1 overexpression is known to induce severe ER stress and activate the UPR in oligodendrocytes, thereby impairing differentiation and leading to myelination defects [14,15,16]. Given its established anti-oxidant, anti-inflammatory, and neuroprotective effects against intracellular stress in cell types [19,20,21,22], Chinpi may attenuate this pathological cascade and protect oligodendrocytes from PLP1-induced cellular stress.

A key molecular finding of this study was the restoration of Akt kinase phosphorylation following treatment with Chinpi. The Akt signaling pathway plays a central role in oligodendrocyte morphological differentiation and myelination through regulation of myelin protein translation/expression [28,29]. In vitro, PLP1 overexpression suppressed Akt phosphorylation and reduced expression of key myelin proteins, including MBP and CNPase, resulting in impaired morphological differentiation. Importantly, treatment with Chinpi extract reversed these abnormalities in a concentration-dependent manner, restoring Akt activation and myelin protein expression. These findings suggest that Chinpi may promote recovery of the Akt signaling cascade, thereby enabling oligodendrocyte precursor cells to overcome differentiation defects caused by PLP1 overexpression.

Crucially, the protective effects observed at the cellular level translated into phenotypic improvements in PLP1 Tg mice, an established in vivo model that recapitulates key features of PMD [27]. In 4-month-old PLP1 Tg mice, oral administration of Chinpi restored reduced expression levels of MBP and CNPase across multiple brain regions, including the cerebral cortex, corpus callosum, and cerebellum. This neuropathological improvement was associated with a significant amelioration of progressive motor dysfunction, as demonstrated by multiple behavioral assessments. In the rotarod and wire hang tests, Chinpi increased the latency to fall, indicating improved motor coordination, balance, and muscle strength. In the tail suspension test, the characteristic hindlimb clasping phenotype, a marker of motor dysfunction, was alleviated, resulting in a wider hindlimb extension angle. Furthermore, in the grid-walking test, Chinpi significantly reduced hindlimb slips, indicating improved sensorimotor coordination.

In conclusion, the present study suggests that Chinpi has the potential to ameliorate PLP1-induced oligodendrocyte differentiation defects and subsequent myelin marker protein expression failure, ultimately improving motor performance at the whole-organism level. Given that Chinpi is a widely used natural product with a favorable safety profile, it may represent a potential pharmacological approach for PMD. Future studies are needed to identify the specific active compounds within Chinpi, including hesperetin and other flavonoids, such as nobiletin and heptamethoxyflavone [36,37,38,39], that contribute to these effects and to clarify their precise molecular targets and underlying mechanisms of action.

### Limitation of This Study

Although the present study demonstrated protective effects of Chinpi in both cellular and mouse models of PMD, several limitations should be acknowledged.

First, Chinpi is a complex natural extract containing multiple bioactive constituents, and the specific constituents responsible for the observed effects remain unclear. Although flavonoids such as hesperetin, nobiletin, and heptamethoxyflavone may contribute to these beneficial effects, further studies using purified compounds will be necessary to identify the major active components and their molecular targets.

## 4. Materials and Methods

### 4.1. Primary and Secondary Antibodies

The following primary antibodies were purchased: mouse monoclonal anti-myelin basic protein (MBP) (Cat. No. 05-675 and Lot No. 2470906; immunoblotting [IB], 1/1000), mouse monoclonal anti-2′, 3′-cyclic nucleotide 3′-phosphodiesterase (CNPase) (Cat. No. C5922 and Lot No. 070M4839; IB, 1/2000), mouse monoclonal anti-phosphorylated Akt kinase (p[S473]Akt) (Cat. No. 4060S and Lot No. 27, IB, 1/1000), and mouse monoclonal anti-Akt kinase (Cat. No. 4691T and Lot No. 28, IB, 1/1000) from Cell Signaling Technology (Danvers, MA, USA); and mouse monoclonal anti-β-actin (Cat. No. 010-27841 and Lot No. WTJ1771; IB, 1/1000) from Fujifilm (Fujifilm, Tokyo, Japan). The following secondary antibodies were purchased: anti-rabbit or mouse IgG F(ab’) conjugated with horseradish peroxidase (Cat. No. 458 and Lot No. 351 or Cat. No. 330 and Lot No. 365; IB, 1/4000) from MBL (Medical & Biological Laboratories, Tokyo, Japan).

### 4.2. Plasmids

The plasmid encoding human PLP1 was generated by GenScript (Piscataway, NJ, USA) (pcDNA3.1(+)-c-eGFP-human PLP1 [Amp^r^], Cat. No. J908Z095G0-2 and Lot No. TK797353). The pCMV5 vector, which shared key vector elements with pcDNA3.1(+), was used as the control vector.

### 4.3. Crossbreeding of Genetically Modified Mice

PLP1 transgenic mice (B6D2-Tg (Plp1) Kmik/Rbrc [Riken No. RBRC00999]), as the PMD model mice, were kindly provided by Riken Bioresource Bank (Saitama, Japan) and were identified by genomic polymerase chain reaction according to the RBRC00999 protocol. Heterozygous mice were fertile until approximately 6 months under standard breeding conditions. Male mice were used for experiments.

### 4.4. Behavior Tests

#### 4.4.1. Tail Suspension Test

Mice were suspended by their tails from the top of the test chamber with their heads approximately 25 cm above the bottom of the chamber. Their behavior was recorded for 2 min using an iPhone. To assess motor function, the angles formed between the bilateral hindlimbs were measured at four time points, 30 s apart, after the start of the test.

#### 4.4.2. Rotarod Test

Mice were placed on a rotarod apparatus, and the rod was accelerated from 4 rpm to 40 rpm over 3 min. Latency was measured when the mouse stopped walking on the rod, either by falling off or by clinging to the rod for two consecutive revolutions. The mean latency from eight independent trials was calculated for each mouse. The training day consisted of four trials with approximately 4 h of rest between trials.

#### 4.4.3. Wire Hang Test

Mice were placed onto a 44.5 cm × 34.5 cm square grid that was inverted 30 cm above a cushioned surface. The mean latency to fall from the grid was calculated from four independent trials for each mouse.

#### 4.4.4. Grid-Walking Test

Mice were placed on an elevated wire grid (40 cm × 20 cm square with 20 mm × 20 mm grids, positioned 30 cm above the floor) and allowed to explore freely for 5 min. Behavior was recorded using an iPhone. Hindlimb foot-fault errors were scored when a limb slipped below the grid surface. The foot-fault rate was calculated by dividing the number of foot-fault errors by the number of hindlimb steps.

### 4.5. Cell Line Culture and Transfection

The FBD-102b cell line, a mouse oligodendroglial precursor cell line, was kindly provided by Dr. Yasuhiro Tomooka and cultured in Dulbecco’s modified Eagle medium (DMEM)/Han’s F-12 nutrient mixed medium (Fujifilm, Tokyo, Japan). FBD-102b cells were cultured on cell culture dishes (Thermo Fisher Scientific, Waltham, MA, USA) in DMEM containing 10% heat-inactivated FBS and PenStrep in 5% CO_2_ at 37 °C. Cells were transfected with the respective plasmids using a Screenfect transfection kit (Fujifilm, Tokyo, Japan). The medium was generally replaced 4 h after transfection. Cells were further cultured for another 44 h and then used for experiments. In each experiment, including those with added Chinpi, less than 5% of the attached cells incorporated trypan blue.

### 4.6. Tissue or Cell Lysis and Immunoblotting

Brain tissues or cells were lysed in RIPA buffer (50 mM Tris-HCl, pH 7.6, 150 mM NaCl, 0.5% sodium deoxycholate, and protease inhibitor cocktail) containing biochemical detergents (1% NP-40 and 0.1% SDS; Nacalai Tesque) (Kyoto, Japan). Unless otherwise indicated, all biochemical experimental steps were performed at 4 °C.

Proteins (10 μg/sample) from cell supernatants were denatured, subjected to gels, and blotted onto polyvinylidene difluoride (PVDF) transfer membranes (Merck Millipore, Darmstadt, Germany). Membranes were blocked with Blocking One reagent (Nacalai Tesque) and immunoblotted using primary antibodies followed by peroxidase-conjugated secondary antibodies. Bound antibodies were detected using ImmunoStar Zeta reagent (Fujifilm). Exposed X-ray films were captured as TIFF image files using a CanoScan LiDE 400 scanner and CanoScan (https://canon.jp/support/software/, accessed on 19 Janurary 2025) (Canon, Tokyo, Japan) software ver. OS14 (Canon). Images in figures are representative of 3 to 6 independent experimental samples. Pixel values for immunoreactive bands were measured using the segment analysis mode of UN-SCAN-IT software ver. 2 (Silk Scientific) (Silk Scientific Inc., Provo, UT, USA). The pixel values of immunoreactive bands were compared to the control values and described as percentages.

### 4.7. Liquid Chromatography–Orbitrap-Mass Spectrometric Analysis of Chinpi Extract

For liquid chromatography–Orbitrap-mass spectrometric (LC-Orbitrap-MS) analysis, 15 mg of Chinpi was extracted with 1.5 mL of 75% ethanol (ethanol:water = 3:1, *v*/*v*) by vortex mixing for 30 min. The extract was centrifuged, and the resulting supernatant was collected and used for LC–Orbitrap-MS analysis. Chinpi extract was analyzed using a Vanquish Flex UHPLC system coupled to an Orbitrap Exploris 120 mass spectrometer equipped with a heated electrospray ionization (H-ESI) source. Chromatographic separation was performed using two Eclipse Plus C18 columns (2.1 mm × 150 mm, 1.8 μm; Agilent Technologies (Santa Clara, CA, USA)) connected in series. Mobile phase A consisted of 0.1% formic acid in water (purified water: formic acid = 1000:1, *v*/*v*), and mobile phase B consisted of 0.1% formic acid in acetonitrile. The flow rate was maintained at 0.30 mL/min from 0 min to 105 min and increased to 0.50 mL/min from 105 min to 118 min. The gradient program was as follows: 2% B at 0 min, increased to 27% B at 60 min and 95% B at 105 min, held at 95% B until 118 min, and then returned to 2% B at 118.1 min followed by re-equilibration. The H-ESI source settings were as follows: spray voltage, 3.5 kV in positive ion mode and 2.5 kV in negative ion mode; sheath gas, 50 arb; auxiliary gas, 10 arb; sweep gas, 1 arb; ion transfer tube temperature, 325 °C; and vaporizer temperature, 350 °C. Full-scan mass spectra were acquired over an *m*/*z* range of 130–1950 at a resolution of 60,000. Data-dependent acquisition was performed using a ddMS^2^ method with four dependent scans per cycle. MS/MS spectra were acquired using higher-energy collisional dissociation at normalized collision energies of 10, 30, and 50%, with a dynamic exclusion duration of 6 s. Putative constituent annotation was performed based on accurate mass measurements and MS/MS fragmentation patterns (Table S1). Candidate compounds were assigned with reference to the Dictionary of Natural Products database (Japan Association for International Chemical Information, Tokyo, Japan) together with literature reports on constituents of *Citrus unshiu* peel. The component analysis in this study was not a non-targeted analysis aimed at identifying unknown compounds; rather, it was conducted to confirm the presence of previously reported major constituents in the Chinpi used. Consequently, compound annotation was performed based on the Dictionary of Natural Products database and the existing literature. The Dictionary of Natural Products was selected for this purpose because it provides comprehensive information regarding source plants and previously reported constituents, making it suitable for evaluating the components of Chinpi.

We used dried peel of *Citrus unshiu* Markowicz conforming to the Japanese Pharmacopeia (JP XVIII) and verified that it meets the specified standards, including hesperidin content (≥4.0% on a dry-weight basis), loss on drying (≤13.0%), ash content (≤4.0%), dilute ethanol-soluble extract content (≥30.0%), and essential oil content (≥0.2 mL per 50.0 g of crude drug powder). The same batch of extract powder of Chinpi was used for both in vitro assays, in vivo studies, and UHPLC-MS analyses.

### 4.8. Preparation and Administration of Dried Citrus Peel

Chinpi used in this study was a crude drug extract powder obtained by hot-water extraction of *Citrus unshiu* peel followed by freeze-drying, manufactured by Tsumura & Co. (Ibaraki, Japan) [40]. Chinpi was used at concentrations ranging from 0.01% to 0.1% (*w*/*v*) for cell-based experiments and at 0.5% (*w*/*v*) for mouse experiments. The Chinpi solution was maintained in standard clear water bottles and replaced every three days. It was administered to the mice for six weeks, during which they had free access to the solution. Since no obvious abnormalities were observed in the mice following the administration of Chinpi at the doses used in this study, the dosages are considered safe [41]. In addition, previous studies have reported that, following the single oral administration of Kampo medicines(Tsumura & Co., Ibaraki, Japan) containing Chinpi, the unchanged forms of hesperidin, narirutin, nobiletin, and heptamethoxyflavone are detected in the blood, with nobiletin and heptamethoxyflavone also being detected in the brain [42,43]. These findings suggest that multiple Chinpi constituents with varying structures and polarities can enter the body following oral administration.

### 4.9. Statistical Analysis

Values are presented as means ± standard deviations from independent experiments. Intergroup comparisons were performed using an unpaired *t*-test with Student’s or Welch’s correction (ver. 2019, Microsoft Corp., One Microsoft Way, Redmond, WA, USA). For comparisons involving more than three groups, one-way analysis of variance (ANOVA) or two-way ANOVA was followed by Bonferroni’s protected least significant difference test as a post hoc comparison using StatPlus software ver. 2022 (AnalystSoft, Alexandria, VA, USA). Differences were considered significant when *p* < 0.05. All experiments and analyses were performed in a blinded manner.

### 4.10. Ethics Statement

Genetically modified and unmodified rodents were cared for in accordance with a protocol (Nos. Y2026-022 and Y2026-023) approved by the Japanese National Research Institute of Child Health and Development Animal Care Committee. Gene recombination and related techniques were performed in accordance with a protocol (Nos. 05-3 and 15-3 or Nos. LS28-30 and LSR3-011) approved by the Japanese National Research Institute of Child Health and Development Gene Recombination Committee or by the Tokyo University of Pharmacy and Life Sciences Gene and Animal Care Committee.

## Data Availability

The datasets used and/or analyzed for the current study are available from the corresponding author upon reasonable request.

## Supplementary Materials

The following supporting information can be downloaded at: https://www.mdpi.com/article/10.3390/ijms27198732/s1.
