# Supplementary material for: Ameliorative Effects of Citrus Unshiu Peel Extract in Cellular and Mouse Models of Pelizaeus–Merzbacher Disease
Source: Int J Mol Sci. 2026 Sep 30;27(19):8732. doi: 10.3390/ijms27198732 (PMC13665114; doi:10.3390/ijms27198732)
Supplement: Supplementary file 1 [file ijms-27-08732-s001.zip › PLP1 Supplemental Figure legends.pdf]

Supplemental figure legends

Figure S1. Overexpression of PLP1 increases ER stress signals. (A-D) FBD-102b cells were transfected with a plasmid encoding PLP1 or an empty vector and differentiated for 5 days. Cell lysates prepared 5 days after induction of differentiation were subjected to immunoblotting using antibodies against phosphorylated eIF2alpha, eIF2alpha, CHOP, and the control GAPDH proteins. Quantification of each immunoreactive band intensity normalized to the loading control is shown (**, $p<0.01$; Student's *t*-test; n = 3 blots). Individual values are plotted as closed circles.

Figure S2. Overexpression of PLP1 decreases Akt activation (A-D) FBD-102b cells were transfected with a plasmid encoding PLP1 or an empty vector and differentiated for 5 days. Cell lysates prepared 5 days after induction of differentiation were subjected to immunoblotting using antibodies against phosphorylated Akt and Akt proteins. Quantification of each immunoreactive band intensity normalized to the loading control Akt are shown (**, $p < 0.01$; Student's *t*-test; n = 5 blots). Individual values are plotted as closed circles.

Figure S3. Treatment with Chinpi decreases increased ER stress signals. (A-D) FBD-102b cells were transfected with a plasmid encoding PLP1 and differentiated for 5 days in the presence or absence of Chinpi extract. Cell lysates prepared 5 days after induction of

differentiation were subjected to immunoblotting using antibodies against phosphorylated eIF2alpha, eIF2alpha, CHOP, and the control GAPDH proteins. Quantification of each immunoreactive band intensity normalized to the loading control is shown (**, $p < 0.01$; Student's *t*-test; n = 3 blots). Individual values are plotted as closed circles.

Figure S4. Treatment with Chinpi increases decreased Akt activation. (A-D) FBD-102b cells were transfected with a plasmid encoding PLP1 and differentiated for 5 days in the presence or absence of Chinpi extract. Cell lysates prepared 5 days after induction of differentiation were subjected to immunoblotting using antibodies against phosphorylated Akt and Akt proteins. Quantification of each immunoreactive band intensity normalized to the loading control Akt are shown (**, $p < 0.01$; Student's *t*-test; n = 3 blots). Individual values are plotted as closed circles.

Figure S5. Computer-saved images of Figure 1.

Figure S6. Computer-saved images of Figure 2.

Figure S7. Computer-saved images of Figure 3.

Figure S8. Computer-saved images of Figure S1.

Figure S9. Computer-saved images of Figure S2.

Figure S10. Computer-saved images of Figure S3.

Figure S11. Computer-saved images of Figure S4.
