# Supplementary material for: Ameliorative Effects of Citrus Unshiu Peel Extract in Cellular and Mouse Models of Pelizaeus–Merzbacher Disease
Source: Int J Mol Sci. 2026 Sep 30;27(19):8732. doi: 10.3390/ijms27198732 (PMC13665114; doi:10.3390/ijms27198732)
Supplement: Supplementary file 1 [file ijms-27-08732-s001.zip › PLP1 Supplemental Table legend.pdf]

Supplemental Table legend

Table S1. Constituents of Chinpi extract. Compounds were putatively annotated based on accurate mass and MS/MS fragmentation patterns. Structural isomers could not be unambiguously distinguished under the analytical conditions used and are therefore reported as putative isomers. Retention time values were obtained from ESI+ analysis when available; otherwise, RT values from ESI− analysis were used. Area (%) values were calculated as the percentage of the total peak area of all filtered features in each ionization mode. Because ionization efficiencies differ among compounds and between ionization modes, these values provide only a semi-quantitative indication of relative signal abundance within each mode and should not be interpreted as absolute concentrations or directly compared across ionization modes.
