# Supplementary material for: Ameliorative Effects of Citrus Unshiu Peel Extract in Cellular and Mouse Models of Pelizaeus–Merzbacher Disease
Source: Int J Mol Sci. 2026 Sep 30;27(19):8732. doi: 10.3390/ijms27198732 (PMC13665114; doi:10.3390/ijms27198732)
Supplement: Supplementary file 1 [file ijms-27-08732-s001.zip › PLP1 Table S1.pdf]

Table S1

Compound	RT (min)	Area (%)		Precursor Ion (m/z)	Major Fragment Ion (m/z)
		ESI+	ESI-		
Feruloyl galactaric acid isomer A	21.4	-	1.4	-385.0775	-191.0199, -85.0295
Feruloyl galactaric acid isomer B	23.4	-	2.2	-385.0775	-191.0198, -85.0295
Feruloyl galactaric acid isomer C	25.5	-	2.2	-385.0773	-191.0198, -85.0295
Feruloyl galactaric acid isomer D	27.0	-	2.0	-385.0773	-191.0198, -85.0295
Feruloyl galactaric acid isomer E	30.4	-	2.5	-385.0773	-191.0198, -85.0295
-	35.1	-	1.1	-431.1922	-385.1873, -153.0923
Vicenin II	36.5	0.7	1.4	+595.1653, -593.1507	+577.1552
-	38.9	-	1.0	-787.2297	-433.1141, -271.0613
-	45.4	-	2.1	-649.2501	-
Narirutin isomer	48.2	1.7	-	+581.1861	+273.0756
Narirutin	48.8	2.2	10.0	+581.1860, -579.1716	+273.0756, -271.0613
-	50.8	0.8	1.4	+815.2237, -813.2094	+347.0761
-	51.9	-	0.9	-813.2093	-
Hesperidin isomer	52.3	1.2	-	+611.1971	+303.0862
Hesperidin	52.8	3.1	10.7	+611.1970, -609.1824	+303.0862, -301.0719
-	53.5	-	1.5	-651.2662	-
-	58.1	-	1.7	-693.2760	-
-	59.1	-	0.8	-711.2868	-607.2764
Neoponcirin	63.9	0.8	1.4	+595.2017, -639.1928	+287.0913, -285.0770
-	67.5	0.8	-	+887.2804	+419.1335, +389.0866
-	68.8	1.4	1.2	+728.3973, -772.3880	-726.3826, -696.3723
Natsudaidain 3-(4-O-HMG-glucoside)	70.4	5.7	5.3	+725.2282, -723.2136	+419.1335, -417.1192
Demethoxynobiletin isomer A	73.1	1.8	-	+373.1278	+343.0811
Nobiletin isomer A	74.6	1.1	-	+403.1382	+373.0914
-	75.2	-	1.4	-515.1920	-469.1868
-	75.3	1.4	-	+343.1173	+313.0706
Demethoxynobiletin isomer B	75.3	1.7	-	+373.1278	-
Nobiletin isomer B	76.9	1.0	-	+403.1382	+373.0915
Nobiletin	77.6	10.1	-	+403.1382	+373.0915
3,3',4',5,6,7,8-Heptamethoxyflavone	79.1	14.0	-	+433.1489	+403.1019
Tangeretin	79.8	4.9	-	+373.1278	+343.0810
Natsudaidain	79.9	1.5	-	+419.1332	+389.0865
Nobiletin isomer C	81.7	0.7	-	+403.1385	+373.0916
